# Proteomic analysis of degradation ubiquitin signaling by ubiquitin occupancy changes responding to 26S proteasome inhibition

**DOI:** 10.1186/s12014-020-9265-x

**Published:** 2020-01-25

**Authors:** Ventzislava Hristova, Shisheng Sun, Hui Zhang, Daniel W. Chan

**Affiliations:** Department of Pathology, Johns Hopkins University, Baltimore, MD 21231 USA

**Keywords:** Ubiquitination, 26S proteasome-mediated degradation, Ubiquitin occupancy, HER2, Quantitative SILAC LC–MS/MS

## Abstract

**Background:**

Ubiquitination is a post-translational modification where ubiquitin is covalently attached to lysine residues on substrate proteins to signal their degradation by the 26S proteasome or initiate other non-degradation functions such as cellular trafficking. The diversity of ubiquitin modifications can be attributed to the variable number of ubiquitin molecules attached to a lysine residue (mono- vs. poly-ubiquitin chains), the type of covalent linkages within poly-ubiquitin chains and the number of lysine residues on a substrate that are occupied by ubiquitin at any given time. The integral role ubiquitination plays in cell homeostasis is reflected by the multitude of diseases associated with impaired ubiquitin modification, rendering it the focus of extensive research initiatives and proteomic discovery studies. However, determining the functional role of distinct ubiquitin modifications directly from proteomic data remains challenging and represents a bottleneck in the process of deciphering how ubiquitination at specific substrate sites impacts cell signaling.

**Methods:**

In this study SILAC coupled with LC–MS/MS is used to identify ubiquitinated proteins in SKOV3 ovarian cancer cells, with the implementation of a computational approach that measures relative ubiquitin occupancy at distinct modification sites upon 26S proteasome inhibition and uses that data to infer functional significance.

**Results:**

In addition to identifying and quantifying relative ubiquitin occupancy at distinct post-translational modification sites to distinguish degradation from non-degradation signaling, this research led to the discovery of nine ubiquitination sites in the oncoprotein HER2 that have not been previously reported in ovarian cancer. Subsequently the computational approach applied in this study was utilized to infer the functional role of individual HER2 ubiquitin-modified residues.

**Conclusions:**

In summary, the computational method, previously described for glycosylation analysis, was used in this study for the assessment of ubiquitin stoichiometries and applied directly to proteomic data to distinguish degradation from non-degradation ubiquitin functions.

## Background

Ubiquitin post-translational modification (PTM) is comprised of a series of enzymatic steps that facilitate the covalent attachment of ubiquitin to specific lysine residues on substrate proteins, which may have one or more ubiquitination sites [1]. Furthermore, ubiquitination can be monomeric or polymeric depending on the number of ubiquitin molecules conjugated to each modified lysine [1–3]. Poly-ubiquitination has diverse conformations that are dependent on the position of the lysine residue within ubiquitin that is covalently bound by the subsequent ubiquitin molecule in the polymer, with chain linkage dictating the functional role of the modification [1–3]. The type of ubiquitination (mono vs. poly) and the linkages within poly-ubiquitin chains are determined by the ubiquitin ligase (E3) enzyme, which is responsible for substrate specificity and recognition of the modification site. Hundreds of E3 enzymes, belonging to different classes of ligases, are currently known and each recognizes specific proteins whose ubiquitination pattern is highly individualized [4]. The high degree of substrate specificity pertaining to ubiquitin modification and the possibility for multiple modification sites within the same protein that can be variably occupied at any given time, contribute to the challenges of characterizing the ubiquitinome of a model system.

Ubiquitination is primarily associated with signaling protein degradation by the 26S proteasome, however ubiquitin modification comes in various forms and many are responsible for non-degradation functions such as receptor internalization and protein trafficking [2]. Differentiating degradation from non-degradation ubiquitin signaling is crucial for understanding the functional role of the modification, however this is a labor-intensive task that requires complex follow-up experiments. A common approach for characterizing ubiquitinated species is to identify the target lysine on the substrate and then determine if the modification occurs via mono-ubiquitination, poly-ubiquitin chains or a combination of both. Advancements in mass spectrometry techniques with respect to improved sensitivity and throughput capabilities have led to large-scale proteomic identification of ubiquitinated proteins, generating vast ubiquitinome databases across species and model systems that hold tremendous potential for assessing ubiquitin signaling [5–10]. This has in turn lead to recent initiatives focused on the development of quantitative methods for the stoichiometric analysis of ubiquitination. For example, Li et al. recently reported a chemical-based quantitative proteomic strategy (IBAQ-Ub) that incorporates derivatization of unmodified lysine residues and stable isotopic labeling of GG remnant motifs on ubiquitin-modified lysines, which in turn allows direct stoichiometric quantification of ubiquitin PTM sites based on MS intensities [9]. In a separate study, Ordureau et al. introduced a parallel reaction monitoring (PRM) targeted approach for the quantification of ubiquitin stoichiometries on peptides resulting from parkin mediated ubiquitination [10]. This technique focused on already identified ubiquitination substrates and used heavy reference peptides corresponding to known ubiquitin modified and unmodified sequences, to quantify substrate ubiquitin modification and abundance in the experimental sample.

Determining the cellular function of distinct ubiquitin modifications with respect to degradation vs. non-degradation signaling remains a major challenge, with most studies focusing on proteins of high interest and overlooking the remainder of the ubiquitinome. To address limitations with the functional assessment of ubiquitination, we apply a computational approach based on previous reports for the determination of absolute stoichiometries by glycosylation and phosphorylation modifications [11, 12]. In our study, we utilize stable isotope labeling with amino acids in cell culture (SILAC) and the SILAC-based analysis described by Sun et al. to characterize ubiquitin stoichiometry by comparing changes in occupied and unoccupied ubiquitination sites on the same lysine among different conditions. Altered ubiquitin occupancy and protein abundance in response to 26S proteasome inhibition are in turn used to infer degradation vs. non-degradation signaling. This technique requires analysis to be performed by SILAC coupled with LC–MS/MS in samples treated with 26S proteasome inhibitor to block ubiquitin-mediated protein degradation. In summary, the proteomic and computational aspects of this method enable rapid identification of ubiquitinated species, determination of ubiquitin stoichiometries at the modification sites and subsequent distinction of degradation vs. non-degradation ubiquitin signaling.

## Methods

### Cell Culture

SKOV3 ovarian carcinoma cells (ATCC HTB-77) originating from the same stock were split into two, one set was cultured in RPMI 1640 media (Gibco) supplemented with 10% FBS (Gibco) and the other in RPMI 1640 media for SILAC (Cambridge Isotope Laboratories) that was supplemented with 10% dialyzed FBS (Cambridge Isotope Laboratories), 120 mg/L ^13^C_6_^15^N_4_l-arginine (Cambridge Isotope Laboratories) and 40 mg/L ^13^C_6_l-lysine (Cambridge Isotope Laboratories). Both SKOV3 cell populations were maintained at the same passage and cultured under the same conditions (37 °C, 5% CO_2_). Incorporation of the isotopically heavy arginine and lysine was allowed to exceed 98% as determined by LC–MS/MS analysis of trypsin digested heavy SKOV3 lysate.

### Proteasome inhibition

Once the l-arginine and l-lysine isotopes were sufficiently incorporated in the protein content of the SKOV3 cells, the cells grown in light RPMI media were treated with 20 μM of proteasome inhibitor, MG132 (Cell Signaling) dissolved in DMSO (Sigma) for 6 h. A parallel experiment was conducted with DMSO treatment to serve as a negative control in place of MG132. Proteasome inhibition by MG132 was confirmed by SDS-PAGE and immunoblotting analysis of lysates from MG132 treated and DMSO control cells using ubiquitin mouse monoclonal antibody (Cell Signaling).

### Cell lysis

Corresponding light and heavy (cultured in ^13^C_6_–^15^N_4_l-arginine and ^13^C_6_l-lysine RPMI) SKOV3 cells at the same passage and confluence were lysed in 8 M urea buffer simultaneously, 6 h after the light cells received 20 μM MG132 or DMSO treatment. Total protein content of the lysate samples was determined using a BCA Protein Assay Kit (Pierce).

### SILAC LC–MS/MS sample preparation

**Fig. 1:**
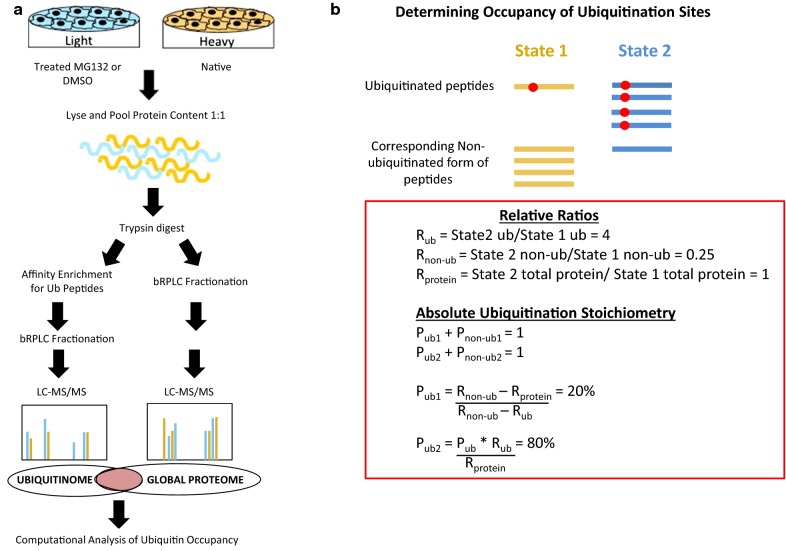
Experimental approach and computational analysis for the assessment of ubiquitin occupancy and total protein ratios. **a** Experimental approach: SILAC LC–MS/MS was used to identify changes in the ubiquitinome of SKOV3 ovarian cancer cells in response to 26S proteasome inhibition by MG132. Cells were cultured in either light or heavy (containing isotopically labeled arginine and lysine residues) RPMI 1640 media. Light cells were treated with either DMSO negative control of MG132 26S proteasome inhibitor, while cells grown in heavy media remained in a native, untreated state. Light and heavy lysates were combined in a 1:1 ratio and after trypsin digestion were either fractionated by bRPLC or ubiquitin-enriched and then fractionated, corresponding to the global and ubiquitinome data sets respectively. Peptides in the global and ubiquitin-enriched samples were detected by LC–MS/MS analysis, which successfully distinguished peptides as originating from the treated (light) or native (heavy) samples based on their m/z ratio. **b** Partially ubiquitinated peptides can exist in one of two forms, ubiquitin occupied or non-ubiquitinated and the percent abundance of both has to equal 100%. Relative ubiquitinated, non-ubiquitinated and protein ratios (Rub, Rnon-ub and Rprotein) were calculated for all partially ubiquitinated peptides in the MG132 treated (State 2) vs. native (State 1) condition. These rations were subsequently used to determine percent ubiquitin occupancy in State 1 (Pub1), which was then utilized for calculating percent ubiquitin occupancy in State 1 (Pub2)

Light and heavy lysates were mixed in a 1:1 ratio based on protein concentration, with 4 mg of each sample combined to give 8 mg of total lysate protein. The mixed sample was then reduced with 10 mM TCEP (Sigma) for 1 h at 37 °C and subsequently alkylated with 12 mM iodoacetamide (Sigma) for 30 min at room temperature. The sample was diluted sixfold with 50 mM Tris HCl pH 8.0 to reduce the urea content and subsequently digested with trypsin overnight at 25 °C using a 1:50 enzyme-to-substrate ratio. Following digestion, the sample was acidified to a final concentration of 1% formic acid and centrifuged at 4° and 1500 g for 15 min to remove precipitated urea and extract the supernatant containing the digested peptides. The supernatant was desalted with reverse-phase SepPak C18 columns (Waters) according to the manufacturer’s guidelines and subsequently the peptide concentration was quantified using BCA assay. A portion of the sample, corresponding to 500 μg of peptides, underwent offline basic reversed phase liquid chromatography (bRPLC) fractionation generating 24 fractions, hereon referred to as global fractions. The peptide concentration of individual global fractions (1–24) was determined via BCA protein assay as μg/μl and based on volume the total peptide content of each fraction was calculated in μg. In accordance with established proteomic procedures, all fractions underwent further desalting and a final drying step to facilitate proper storage. Prior to LC–MS/MS analysis, global fractions were resuspended in 3% acetonitrile/0.1% formic acid using corresponding volumes to generate 1 μg/μl peptide concentrations across all fractions (this was confirmed via BCA analysis). From the remaining desalted sample, 6.5 mg of peptides were subjected to ubiquitin-enrichment with the PTMScan Ubiquitin Remnant Motif Kit (Cell Signaling). Lysine resides modified with ubiquitin retain a Gly–Gly motif (corresponding to the terminal two amino acids of ubiquitin) following trypsin digestion and this K-ɛ-GG ubiquitin remnant is recognized by the affinity purification Cell Signaling Kit [8]. To achieve high efficiency purification of ubiquitinated peptides, preliminary studies were conducted to evaluate the binding affinity and extraction of ubiquitin-modified peptides using variable peptide concentrations and incubation times. This optimization is based on a previously published method for ubiquitin remnant motif enrichment by Udeshi et al. [8] For maximum PTM enrichment efficiency, the sample was divided into four sub-fractions of equal concentration that were representative of the parent sample peptide composition and each one was incubated with 20 μl of PTM antibody slurry, followed by incubation at 4 °C for 2 h with rotation. Extracted ubiquitinated peptides were then pooled and fractionated using the same bRPLC method that was used to generate the global fractions set. Peptide concentration was determined for the ubiquitin-enriched fractions using BCA protein assay and the samples were subsequently desalted and dried. Prior to LC–MS/MS analysis the ubiquitin enriched fractions were resuspended in 3% acetonitrile/0.1% formic acid to give a final peptide concentration of 1 μg/μl (confirmed by BCA). Both global and ubiquitin-enriched fractions were subsequently analyzed by LC–MS/MS on an LTQ-Orbitrap Velos Pro instrument (Thermo Scientific), with 1 μl, corresponding to 1 μg, of each fraction injected for consistency. The distribution of peptides within each fraction is uniform, hence the 1 μl (1 μg) volume used for proteomic analysis is representative of the relative abundance of peptides in that fraction and allows direct comparison among samples (Fig. 1a). Chromatographic separation was performed using a 75 µm × 50 cm Acclaim PrepMap RSLC 2 μm C18 separating column at a flow rate of 0.3 microliters per minute and a multistep gradient with 0.1% formic acid in water (A) and 0.1% formic acid in 95% acetonitrile (B). Chromatographic gradient as follows: 0 min, 4% B; 2 min, 4% B; 10 min, 10% B; 100 min, 35% B; 105 min, 95% B; 115 min, 95% B; 116 min, 4% B; 120 min, 4% B. Data were collected in positive ion mode with the following settings: full scan spectra acquisition time 120 min, full scan range 400–1800 m/z, resolution 60,000, isolation width of 1.0 Th., with a maximum injection time of 10 ms followed by data-dependent HCD MS/MS (30,000 resolution, scan range 400–1800 m/z, collision energy 35, 0.1 ms activation time) of the 10 most abundant ions using an isolation width of 2.0 Th and a minimum signal requirement of 500 to trigger the MS2 scan.

### LC–MS/MS data analysis

Global and ubiquitin-enriched data from two independent, biological replicate SILAC LC–MS/MS experiments was processed using MaxQuant set for ubiquitin remnant motif recognition in conjunction with trypsin cleavage. The light and heavy signal intensities observed for each peptide in the ubiquitinated and non-ubiquitinated form, were used to calculate relative ubiquitinated, non-ubiquitinated and protein ratios (R_ub_, R_non-ub_ and R_protein_) of MG132 treated (light) vs. native (heavy) conditions (Fig. 1b). These ratios were then used to determine ubiquitination stoichiometries, percent ubiquitin occupancy, for distinct peptides under each condition (native and MG132 treated) [11, 12].

Ubiquitinated lysine residues were matched with their corresponding non-ubiquitinated form found in the global data set. Ubiquitin PTM prevents trypsin cleavage after the modified lysine, hence peptides identified as ubiquitinated at C-terminus lysine residues were omitted from further analysis. Additionally, this approach is limited to assessing stoichiometry for residues observed in both the ubiquitin-modified and non-modified form that exhibit partial ubiquitin occupancy. Therefore, peptides observed only in the ubiquitinated state, with no corresponding non-modified form detected in the global data, were excluded from relative occupancy ratio calculations (Additional file 1: Tables S2, S4). Furthermore, in the absence of ubiquitin modification lysine residues are subject to trypsin cleavage. To address the complexity of matching ubiquitinated lysine residues with their corresponding non-modified forms, global proteomic data analysis identified all peptides containing the lysine of interest regardless of peptide length and residue composition. Subsequently, abundance of the non-modified form was determined from all intensities belonging to peptides containing the lysine of interest in the non-ubiquitinated state, regardless if it was at the C-terminus or within the peptide sequence (due to a missed trypsin cleavage).

Changes in ubiquitination and protein abundance between MG132 and native conditions were expressed as relative ubiquitination, non-ubiquitination and protein ratios. The ubiquitination ratio (R_ub_) of MG132 treated to native state corresponds to the light signal intensities vs. heavy signal intensities ratio for each distinct ubiquitinated peptide (Fig. 1b). The non-ubiquitinated relative ratio (R_non-ub_) of MG132 treated vs native state is obtained from the global sample peptides and corresponds to non-ubiquitinated light signal intensities of each peptide in the MG132 sample vs. the corresponding heavy signal intensities of that peptide in the native state (Fig. 1b). Finally, the relative protein ratio (R_protein_) is determined by dividing all light signal intensities for a peptide (both in the ubiquitinated and non-ubiquitinated state) by all heavy signal intensities for the corresponding peptide in the native state. These three relative ratios (R_ub_, R_non-ub_ and R_protein_) are then used to determine ubiquitin occupancy stoichiometries at the distinct ubiquitination sites. Since each lysine can only exist in two states, ubiquitinated or non-ubiquitinated, the percentage of ubiquitinated and non-ubiquitinated species in each state must add up to 100% (Fig. 1b). Hence, non-ubiquitinated stoichiometries for each lysine were calculated by subtracting percent ubiquitin occupancy from 100%. Ubiquitin occupancy stoichiometries in the native state were calculated for each peptide as percent ubiquitination (P_ub1_) using the three relative ratios determined for that peptide. In turn the percent ubiquitin occupancy in the native state was used to determine the percent ubiquitin occupancy in the MG132 condition (P_ub2_) (Fig. 1b). The same computational approach was applied to DMSO treated samples to determine the relative DMSO vs. native ratios and the corresponding ubiquitin occupancy stoichiometries.

## Results

Ubiquitin post-translational modification is highly diverse with respect to the number of ubiquitin molecules bound to a substrate and linkages within poly-ubiquitin chains, all of which determine the functional role of the modification. Ubiquitination can signal cellular trafficking and receptor internalization among other things, but it is most commonly associated with facilitating degradation by the 26S proteasome, which recognizes poly-ubiquitin chains of certain architecture and removes the modified protein. Hence, comprehensive analysis of ubiquitinated proteins can be difficult due to their low abundance and rapid turnover. To overcome this challenge, proteasome inhibitors such as MG132 are routinely used to block ubiquitin-mediated degradation leading to accumulation of ubiquitinated substrates that can be detected through proteomic analysis. In this study, SILAC is coupled with LC–MS/MS to identify ubiquitinated peptides and compare abundance between native (heavy) and MG132 treated (light) SKOV3 ovarian cancer cells (Fig. 1a). This technique relies on the incorporation of isotopic arginine and lysine residues to distinguish peptides originating from each sample (heavy vs. light) and is invaluable for assessing proteome changes under variable conditions such as proteasome inhibition for ubiquitinome analysis. Here, we implement a proteomic approach that has previously been used for the quantification of absolute glycosylation stoichiometries, to determine site-specific ubiquitin stoichiometries and compare changes in relative ubiquitin occupancy ratios at PTM sites between native and MG132 treated samples (Fig. 1b). This approach utilizes SILAC LC–MS/MS data to measure relative ubiquitin occupancy and total protein ratios that are subsequently used to distinguish degradation from non-degradation ubiquitin signaling.

**Fig. 2:**
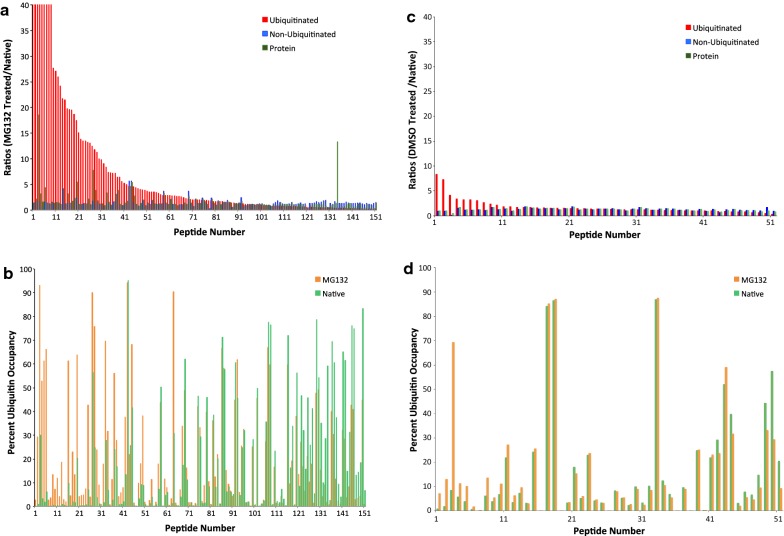
Ubiquitin occupancy, non-ubiquitin occupied and total protein ratios were generated for all partially ubiquitinated peptides detected in MG132 and DMSO control treated samples. **a** Relative ubiquitinated, non-ubiquitinated and protein ratios for all partially ubiquitinated peptides in the MG132 treated vs. native state. **b** Percent ubiquitin occupancy for partially ubiquitinated peptides in the MG132 treated and native conditions. **c** Relative ubiquitinated, non-ubiquitinated and protein ratios for all partially ubiquitinated peptides in the DMSO treated vs. native state. **d** Percent ubiquitin occupancy for partially ubiquitinated peptides in the DMSO treated and native conditions

The premise for this work is that each lysine residue that is subject to ubiquitin modification can exist in one of two states, ubiquitin occupied or unoccupied, with the sum of the two states accounting for 100% of that lysine’s abundance. The MG132 to native ubiquitinated, non-ubiquitinated and protein ratios are calculated for each peptide using the ubiquitin enriched and global data sets (Figs. 1b, 2a, c). In turn, the ratios are used to calculate percent ubiquitin occupancy for each peptide in the native and MG132 or DMSO treated states (Figs. 1b, 2b, d). Subsequently changes in ubiquitination with MG132 or DMSO treatment are expressed as percent ubiquitin occupancy at each peptide (Fig. 2b, d).

Proteins modified for proteasome-mediated degradation evade turnover in the presence of MG132, but not DMSO, and have increased relative ubiquitin and protein ratios as well as ubiquitin occupancy stoichiometries. Initial analysis detected 251 ubiquitinated peptides in the MG132 sample after ubiquitin-enrichment that had both light and heavy signals, of those 151 existed in a partially ubiquitinated state and the remaining 100 were only found in the ubiquitin-modified form and were excluded from the computational analysis (Additional file 1: Tables S1, S2). Ubiquitin-enrichment of the DMSO sample led to the detection of 98 ubiquitinated peptides and of those 51 had lysine PTM sites observed in both the ubiquitinated and non-ubiquitinated form (Additional file 1: Tables S3, S4). Relative ubiquitin occupancy ratios were calculated for each lysine in the treated (light) vs. native (heavy) state (Fig. 2a, c). MG132 treatment led to a dramatic increase in ubiquitin occupancy ratios, reaching as high as 85 (Fig. 2a, Additional file 1: Table S1). The same quantitative analysis for DMSO control treated SKOV3 cells detected only three peptides with a relative ubiquitin occupancy ratio greater than 4, with the maximum being 8.4 (Fig. 2c, Additional file 1: Table S3). The percentage of ubiquitinated and unoccupied lysine PTM sites was subsequently determined for the native, MG132 and DMSO treated samples (Fig. 2). Partially ubiquitinated lysine PTM sites that experience an increase in ubiquitin occupancy with MG132, include those that directly induce protein degradation as well as residues that are indirectly impacted by proteasome activity. To further investigate which residues were modified for degradation signaling, the ubiquitinated and protein ratios were analyzed with respect to the corresponding percent ubiquitin occupancies. A subset of the peptides, whose ubiquitination increased with proteasome inhibition, also experienced increased abundance with MG132 treatment relative to the native state (Fig. 2), indicating that these sites serve as degradation signals. For example, four of the six ubiquitination sites identified in vimentin (*VIME* gene), a protein associated with epithelial-to-mesenchymal transition (EMT) that is upregulated across cancer types, exhibited an increase in ubiquitin occupancy with MG132 indicating these sites are responsible for signaling ubiquitin-mediated degradation of vimentin by the 26S proteasome (Additional file 1: Table S1) [13]. These data and computational analysis are in agreement with reported findings in ovarian epithelial cells showing that vimentin undergoes proteasomal degradation upon ubiquitination by the TRIM56 ubiquitin ligase [14, 15]. Although previous work by Zhao et al. identified TRIM56 as responsible for ubiquitinating vimentin in SKOV3 ovarian cancer cells, the exact ubiquitin-modification sites were not identified and the data presented in this manuscript is the first report of specific lysine residues within vimentin that are ubiquitinated for degradation signaling [15]. Taken independently, this finding holds tremendous potential for therapeutic approaches to target increased vimentin levels in cancer that induce EMT.

Proteins may have multiple ubiquitination sites and when interpreting the results in this study, it is crucial to keep in mind that any combination of ubiquitin occupancies may exist at any given time [1]. Comparing partially ubiquitinated PTM sites between MG132 and DMSO treatment, demonstrated that proteasome inhibition increased percent ubiquitin occupancy to a significantly greater extent than DMSO (Fig. 2 and Additional file 1: Table S1, S3). However, some peptides did not show a change in ubiquitin occupancy with proteasome inhibition (Fig. 2) and these represented ubiquitin modification sites that serve non-degradation functions.

**Fig. 3:**
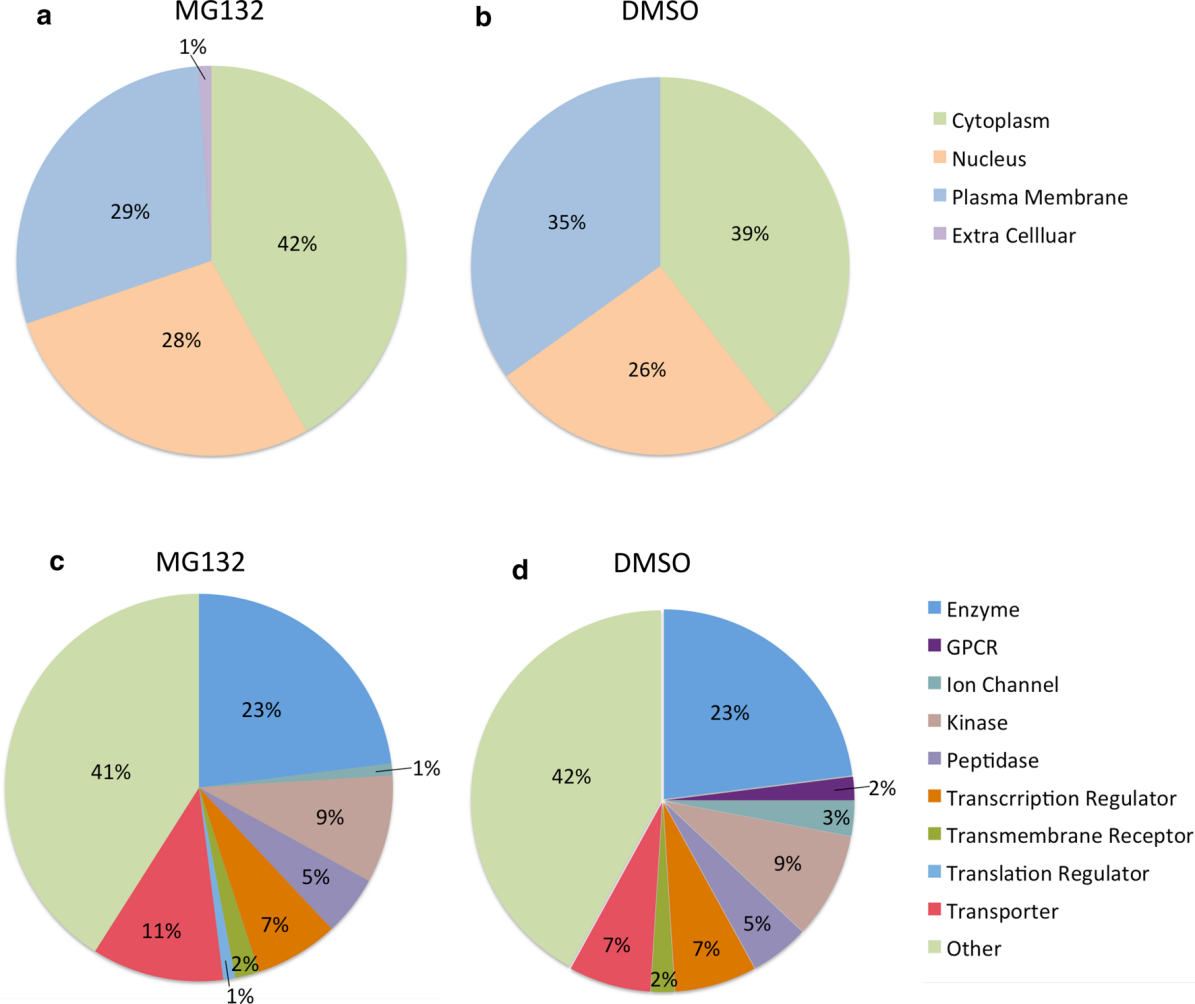
Overview of ubiquitinated proteins identified in SKOV3 ovarian cancer cells with MG132 and DMSO control treatment. **a** Cellular distribution of ubiquitinated proteins identified in MG132 treated SKOV3 cells. **b** Cellular distribution of ubiquitinated peptides observed in DMSO control treated SKOV3 cells. **c** Functional classification of ubiquitinated proteins detected in MG132 treated SKOV3 cells. **d** Functional classification of ubiquitinated proteins detected in DMSO control treated SKOV3 cells

Assessment of the cellular localization of the ubiquitinated proteins identified in this study, showed similar distribution between MG132 and DMSO samples (Fig. 3a, b). Functional analysis of the ubiquitinome focused on broad protein categories and also exhibited a predominantly similar distribution between MG132 and DMSO treatment, with a few differences including increased ubiquitination of transporter proteins and translational regulators with MG132 treatment (Fig. 3c, d). These analysis suggest that in this cell model, MG132 treatment does not disproportionately shift ubiquitin-modification to select protein classes, but primarily stabilizes ubiquitinated species modified for degradation signaling across all categories.

**Fig. 4:**
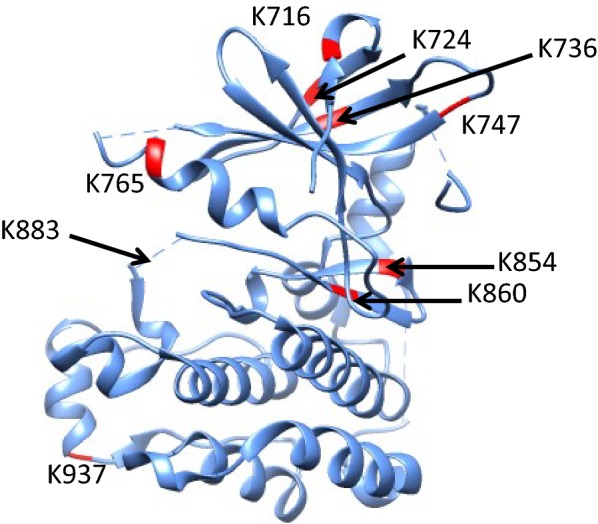
Structural conformation of HER2 intracellular kinase domain and position of lysine residues identified as ubiquitin modification sites in SKOV3 cells. Eight of the nine ubiquitination sites in HER2 are shown in red, the ninth is Lys 883 and is located in the unstructured portion of this model, which cannot be highlighted

Mutations in ubiquitin ligase enzymes and substrates have been reported in numerous cancers, generating a strong interest in the role of ubiquitin signaling in oncology [16–18]. The SKOV3 ovarian cancer ubiquitinome analysis performed here focused on developing a fast approach to quantify ubiquitin occupancy and total protein abundance ratios for distinct modification sites in an effort to rapidly distinguish the functional role as degradation or non-degradation signaling. In addition to addressing these aims, this study led to the exciting discovery of ubiquitin modifications at lysine residues known to be mutated in various cancer types. Of the 151 ubiquitinated proteins that we reported, 91 are known to be mutated across a broad range of cancers. Furthermore, a COSMIC database search identified specific mutations that alter lysine residues identified as ubiquitination sites in this study. Examining both partially ubiquitinated sites and ones detected entirely in the ubiquitin-modified form, we identified 12 lysine residues in 8 distinct proteins that were reported as mutated in cancer patients. The most provocative and exciting results pertain to the oncoprotein HER2 (*ERBB2* oncogene), whose overexpression is widely associated with breast and ovarian cancer. To date there are few reports of HER2 ubiquitination in breast cancer models, which do not provide insight into the functional role of distinct ubiquitin-modified sites [19, 20]. Most importantly, there are no prior reports of HER2 ubiquitin modification in ovarian cancer, where HER2 overexpression and signaling do not respond to targeted therapies that have been successful in the treatment of HER2 positive breast cancer. The discrepancy in response to HER2 targeted therapy in ovarian cancer indicates that the tyrosine kinase is regulated by mechanisms distinct from those in breast tissue. In this study, we identified nine ubiquitination sites within HER2 in SKOV3 ovarian cancer cells (Lys 716, Lys 724, Lys 736, Lys 747, Lys 765, Lys 854, Lys 860, Lys 883 and Lys 937), including a novel ubiquitin modification at lysine 883 previously not reported in breast cancer. All HER2 ubiquitin-modified lysine residues observed in our SILAC LC–MS/MS data set are located within or in close proximity of the tyrosine kinase domain (Fig. 4). The functional role of these ubiquitination sites was assessed by calculating relative ubiquitin and protein ratios for the seven partially ubiquitinated lysines along with percent ubiquitin occupancy (Table 1). Two of the nine lysine residues, at positions 716 and 854, were only detected in a ubiquitin-occupied state and therefore were excluded from the computational analysis (Additional file 1: Table S2). Treatment with MG132 increased ubiquitin occupancy and total protein ratios at residues 724, 736, 765, 860, 883 and 937 with Lys 724 Lys 860, Lys 883 having occupancy ratios greater than ten (Table 1). While lysine 747 did not show elevated ubiquitin occupancy upon proteasome inhibition, with a ratio of 1.65, an increase in peptide abundance was noted by the relative total protein ratio of 1.76. This increase in abundance can be attributed to stabilization of the protein as a whole due to inhibited degradation by other HER2 lysine sites that were simultaneously occupied. The computational analysis performed in this study showed an increase in relative ubiquitin and total protein ratios for HER2 residues Lys 724, Lys 860 and Lys 883 (Table 1), implicating them as degradation signals. This insight into HER2 targeting for removal by the 26S proteasome holds great potential for therapeutic approaches in ovarian cancer.

**Table 1:** Relative ubiquitin occupancy and total protein abundance ratios for the seven partially ubiquitinated sites in HER2

Lysine residue position in HER2	Peptide sequence	Ubiquitinated ratio (Rub)	Protein ratio (Rprotein)
724	VKVLGSGAFGTVYK	13.2	2.18
736	VLGSGAFGTVYKGIWIPDGENVK	3.96	2.02
747	GIWIPDGENVKIPVAIK	1.65	1.76
765	ANKEILDEAYVMAGVGSPYVSR	4.96	1.75
860	SPNHVKITDFGLAR	19.89	3.17
883	LLDIDETEYHADGGKVPIK	11.75	3.85
937	EIPDLLEKGER	4.56	2.78
716	ILKETELR	Only ubiquitin occupied state	
854	NVLVKSPNHVK	Only ubiquitin occupied state	

## Discussion

The functional variability of ubiquitination and the diversity in poly-ubiquitin chain architecture have complicated studies investigating ubiquitin signaling in disease models [2]. Despite advancements in mass spectrometry and proteomic techniques that have led to the generation of continuously expanding ubiquitinome databases, these large data sets cannot conclusively infer the functional significance of distinct ubiquitination sites [5–10]. In this study we outline the application of SILAC LC–MS/MS that allows for the rapid distinction of non-degradation and degradation ubiquitin signaling through SILAC-based computational analysis of proteomic data and assessment of relative ubiquitin occupancy and total protein abundance ratios at distinct PTM sites. Ubiquitin peptide enrichment efficiency from ovarian cancer cells in the MG32 treated and native state as well as the percentage of peptides subjected to LC–MS/MS analysis affect ubiquitinated, non-ubiquitinated and the total protein abundances. Therefore, careful control of these factors and the use of quantitative proteomic analysis for the determination of ubiquitin, non-ubiquitin and protein ratios coupled with the use of isotopic labeling are necessary for the accurate calculation of ubiquitin occupancies. Employing a SILAC-based approach enables quantitative comparison of ubiquitination between different conditions, in this case native and MG132 or DMSO control treated. In turn, inhibiting 26S proteasome activity with MG132 leads to the stabilization and accumulation of ubiquitinated proteins that would otherwise be rapidly degraded and undetectable under native conditions. As expected, our work showed an increase in ubiquitinated peptides with MG132 treatment compared to DMSO and non-treated, native samples (Fig. 2). Comparison of the ubiquitin-enriched and global data sets found that many of the ubiquitinated lysine residues and corresponding proteins were only detected in the ubiquitin-occupied state with no non-ubiquitinated form observed in global data sets. These peptides likely originate from low abundance proteins that are rapidly degraded under native conditions and cannot be detected without blocking proteasome activity. However, as their percent ubiquitin occupancy could not be accurately verified, they were omitted from the computational analysis in this study. In contrast, partially ubiquitinated lysine residues, which were observed as both ubiquitin-modified and non-modified heavy and light peptides, underwent quantitative analysis to calculate relative ubiquitinated, non-ubiquitinated and protein ratios along with percent ubiquitin occupancy for native and MG132 or DMSO treated samples (Fig. 2). As predicted, ubiquitinated ratios and percent ubiquitin occupancy were high for many lysine residues with MG132 treatment compared to native state and DMSO treated, while others did not exhibit a significant difference, indicating that their modification is independent of 26S proteasomal activity. Percent ubiquitin occupancy for individual lysine PTM sites was used to derive the percent of those residues in the non-ubiquitinated form for treated (light) and native (heavy) samples (Figs. 1b, 2). The MG132 treated to native state ratios for each peptide were used to extrapolate the functional role of distinct ubiquitin modifications, with ubiquitination sites that were modified for degradation exhibiting an increase in the relative ubiquitin occupancy and total protein ratios with MG132 treatment compared to native state. Conducting complex in situ mutagenesis experiments for degradation and non-degradation signaling to demonstrate proof of principle is beyond the scope of this study. However, we utilize previously published work, when available, to support this computational approach as evidenced by the ubiquitin-mediated degradation of vimentin and the correspondingly high ubiquitin-occupancy and total peptide ratios observed in our data [14, 15]. Hence this SILAC-based LC–MS/MS computational method, which has previously been applied for glycosylation stoichiometry analysis, serves as a rapid technique for distinguishing ubiquitin function as degradation vs. non-degradation signaling [11, 12].

Additionally, this study led to the serendipitous finding of nine ubiquitination sites within the HER2 oncoprotein in ovarian cancer SKOV3 cells (Table 1). This intriguing discovery identified novel HER2 ubiquitin modifications such as that on Lys 883, as well as HER2 ubiquitination at lysine residues previously reported in breast cancer. Interestingly all HER2 ubiquitination sites are located within or in very close proximity to the kinase domain, suggesting potential involvement in the regulation of kinase activity (Fig. 4). Of the nine ubiquitin modified lysine residues in HER2, two were only detected in the ubiquitin occupied state and their relative ubiquitin occupancy could not be confirmed with certainty. However, the remaining seven ubiquitination sites had varying degrees of ubiquitin occupancy ratios, with several showing increased ubiquitination and protein abundance in response to MG132, indicating those sites play a role in HER2 degradation signaling (Table 1). A detailed review of cancer mutations using COSMIC, identified Lys 716, Lys 724 and Lys 937 as HER2 mutations prevalent in various cancer types. Furthermore, computational data from this study implicated ubiquitination at Lys 724 and Lys 716 as degradation signals that when impaired can lead to protein accumulation, which is a hallmark of HER2 positive ovarian and breast cancer. The characterization of HER2 ubiquitination sites that facilitate HER2 degradation in a proteasome-dependent manner is a provocative finding that can very well lead to innovations in targeted therapy [21]. Given that HER2 positive ovarian cancer patients show limited response to targeted therapies that are successful in the treatment of HER2 positive breast cancer, these findings can be utilized in developing ovarian carcinoma specific therapeutics [22–26]. To date there are few reports of HER2 ubiquitination, all in breast cancer models, and the functional role of distinct modifications as well as the mechanisms that initiate ubiquitination at specific lysine residues remain to be determined. Therefore, the computational approach utilized in this manuscript and the subsequent relative ubiquitin occupancy and protein abundance data represent an early step towards understanding HER2 ubiquitin regulation in ovarian cancer and how it may translate to new, effective therapies.

## Conclusions

Interpreting the functional outcome of highly complex ubiquitin post-translational modifications can be a daunting and time-consuming task that is essential to understanding how cell signaling is regulated by ubiquitination. In this study, a SILAC LC–MS/MS approach was successfully applied to detect ubiquitinated peptides and subsequent computational analysis of the proteomic data were used to calculate percent ubiquitin occupancy and relative ubiquitin occupancy and protein abundance ratios for distinct lysine residues with the goal of rapidly identifying proteins destined for ubiquitin-mediated proteasomal degradation. In turn, lysine residues whose ubiquitin occupancy and corresponding total protein ratios did not change in response to MG132 proteasome inhibitor, were designated as serving a non-degradation function. The utility of SILAC LC–MS/MS for the computational assessment of prost-translational modification occupancies has been applied in the past to phosphorylation and glycosylation analysis [11, 12]. Here the SILAC-based method previously described for glycosylation stoichiometry analysis, is used to establish relative ubiquitin occupancy ratios for distinct lysine restudies with the additional advantage that computational data analysis can rapidly distinguishing degradation from non-degradation ubiquitin signaling.

## Supplementary information

**Additional file 1: Table S1.** Ubiquitin occupancy of partially ubiquitinated peptides detected in the ubiquitinated and non-ubiquitin modified form in SKOV3 ovarian cancer cells after MG132 treatment. **Table S2.** Ubiquitinated peptides that are only detected in the modified state in ubiquitin-enriched samples following MG132 treatment of SKOV3 cells. **Table S3.** Ubiquitin occupancy of partially ubiquitinated peptides identified in the DMSO control treated sample. **Table S4.** Peptides only detected as ubiquitinated after DMSO treatment, with no corresponding non-ubiquitinated form in the global DMSO data set

## Data Availability

The datasets used and/or analyzed during the current study are available from the corresponding author on reasonable request.

## Abbreviations

SILAC: stable isotope labeling with amino acids in cell culture
LC–MS/MS: liquid chromatography tandem mass spectrometry
bRPLC: basic reversed phase liquid chromatography
HER2: human epidermal growth factor receptor 2
PTM: post-translational modification
EMIT: epithelial-to-mesenchymal transition
COSMIC: Catalogue of Somatic Mutations in Cancer
E3: ubiquitin ligase enzyme
Gly: glycine amino acid
Lys: lysine amino acid
FBS: fetal bovine serum
DMSO: dimethyl sulfoxide
TCEP: tris x(2-carboxyethyl)phosphine
BCA: bicinchoninic acid (protein assay)
M: molar
µM: micro molar
m/z: mass to charge ratio

## References

[1] Behrends C, Harper JW (2011). Constructing and decoding unconventional ubiquitin chains. Nat Struct Mol Biol.

[2] Akutsu M, Dikic I, Bremm A (2016). Ubiquitin chain diversity at a glance. J Cell Sci.

[3] Yau R, Rape M (2016). The increasing complexity of the ubiquitin code. Nat Cell Biol.

[4] Metzger MB, Hristova VA, Weissman AM (2012). HECT and RING finger families of E3 ubiquitin ligases at a glance. J Cell Sci.

[5] Ordureau A, Munch C, Harper JW (2015). Quantifying ubiquitin signaling. Mol Cell.

[6] Rose CM, Isasa M, Ordureau A, Prado MA, Beausoleil SA, Jedrychowski MP, Finley DJ, Harper JW, Gygi SP (2016). Highly multiplexed quantitative mass spectrometry analysis of ubiquitylomes. Cell Syst..

[7] Wagner SA, Beli P, Weinert BT, Nielsen ML, Cox J, Mann M, Choudhary C (2011). A proteome-wide, quantitative survey of in vivo ubiquitylation sites reveals widespread regulatory roles. Mol Cell Proteomics..

[8] Udeshi ND, Mertins P, Svinkina T, Carr SA (2013). Large-scale identification of ubiquitination sites by mass spectrometry. Nat Protoc.

[9] Li Y, Evers J, Luo A, Erber L, Postler Z, Chen Y (2019). A quantitative chemical proteomics approach for site-specific soichiometry analysis of ubiquitination. Angew Chem Int Ed Engl.

[10] Ordureau A, Paulo JA, Zhang W, Ahfeldt T, Zhang J, Cohn EF, Hou Z, Heo J, Rubin LL, Sidhu SS, Gygi SP, Harper JW (2018). Dynamics of PARKIN-dependent mitochondrial ubiquitylation in induced-neurons and model systems revealed by digital snapshot proteomics. Mol Cell.

[11] Sun S, Zhang H (2015). Large-scale measurement of absolute protein glycosylation stoichiometry. Anal Chem.

[12] Wu R, Haas W, Dephoure N, Huttlin EL, Zhai B, Sowa ME, Gygi SP (2011). A large-scale method to measure absolute protein phosphorylation stoichiometries. Nat Methods.

[13] Mendez MG, Kojima S, Goldman RD (2010). Vimentin induces changes in cell shape, motility, and adhesion during the epithelial to mesenchymal transition. FASEB J.

[14] Zhao L, Zhang P, Su XJ, Zhang B (2018). The ubiquitin ligase TRIM56 inhibits ovarian cancer progression by targeting vimentin. J Cell Physiol.

[15] Zhao L, Wang ZG, Zhang P, Yu XF, Su XJ (2019). Poly r(C) binding protein 1 regulates posttranscriptional expression of the ubiquitin ligase trim56 in ovarian cancer. IUBMB Life.

[16] Lipkowitz S, Weissman AM (2011). RINGs of good and evil: RING finger ubiquitin ligases at the crossroads of tumour suppression and oncogenesis. Nat Rev Cancer.

[17] Morris JR, Solomon E (2004). BRCA1: BARD1 induces the formation of conjugated ubiquitin structures, dependent on K6 of ubiquitin, in cells during DNA replication and repair. Hum Mol Genet.

[18] Nakayama KI, Nakayama K (2006). Ubiquitin ligases: cell-cycle control and cancer. Nat Rev Cancer.

[19] Mertins P, Mani DR, Ruggles KV, Gillette MA, Clauser KR, Wang P, Wang X, Qiao JW, Cao S, Petralia F, Kawaler E, Mundt F, Krug K, Tu Z, Lei JT, Gatza ML, Wilkerson M, Perou CM, Yellapantula V, Huang KL, Lin C, McLellan MD, Yan P, Davies SR, Townsend RR, Skates SJ, Wang J, Zhang B, Kinsinger CR, Mesri M, Rodriguez H, Ding L, Paulovich AG, Fenyo D, Ellis MJ, Carr SA, Nci C (2016). Proteogenomics connects somatic mutations to signalling in breast cancer. Nature.

[20] Marx C, Held JM, Gibson BW, Benz CC (2010). ErbB2 trafficking and degradation associated with K48 and K63 polyubiquitination. Cancer Res.

[21] Cromm PM, Crews CM (2017). Targeted protein degradation: from chemical biology to drug discovery. Cell Chem Biol.

[22] Teplinsky E, Muggia F (2014). Targeting HER2 in ovarian and uterine cancers: challenges and future directions. Gynecol Oncol.

[23] Bookman MA, Darcy KM, Clarke-Pearson D, Boothby RA, Horowitz IR (2003). Evaluation of monoclonal humanized anti-HER2 antibody, trastuzumab, in patients with recurrent or refractory ovarian or primary peritoneal carcinoma with overexpression of HER2: a phase II trial of the Gynecologic Oncology group. J Clin Oncol.

[24] Makhija S, Amler LC, Glenn D, Ueland FR, Gold MA, Dizon DS, Paton V, Lin CY, Januario T, Ng K, Strauss A, Kelsey S, Sliwkowski MX, Matulonis U (2010). Clinical activity of gemcitabine plus pertuzumab in platinum-resistant ovarian cancer, fallopian tube cancer, or primary peritoneal cancer. J Clin Oncol.

[25] Gordon MS, Matei D, Aghajanian C, Matulonis UA, Brewer M, Fleming GF, Hainsworth JD, Garcia AA, Pegram MD, Schilder RJ, Cohn DE, Roman L, Derynck MK, Ng K, Lyons B, Allison DE, Eberhard DA, Pham TQ, Dere RC, Karlan BY (2006). Clinical activity of pertuzumab (rhuMAb 2C4), a HER dimerization inhibitor, in advanced ovarian cancer: potential predictive relationship with tumor HER2 activation status. J Clin Oncol.

[26] Fleming GF, Sill MW, Darcy KM, McMeekin DS, Thigpen JT, Adler LM, Berek JS, Chapman JA, DiSilvestro PA, Horowitz IR, Fiorica JV (2010). Phase II trial of trastuzumab in women with advanced or recurrent, HER2-positive endometrial carcinoma: a Gynecologic Oncology Group study. Gynecol Oncol.

