# Correction to: Proteomic analysis of degradation ubiquitin signaling by ubiquitin occupancy changes responding to 26S proteasome inhibition

**DOI:** 10.1186/s12014-020-09271-0

**Published:** 2020-02-21

**Authors:** Ventzislava Hristova, Shisheng Sun, Hui Zhang, Daniel W. Chan

**Affiliations:** Department of Pathology, Johns Hopkins University, Baltimore, MD 21231 USA

## Correction to: Clin Proteom (2020) 17:2 10.1186/s12014-020-9265-x

In the original publication of the article, Figure 2d was published incorrectly. The corrected Fig. 2 was given in this Correction article [1]. The original article has been corrected.

**Fig. 2:**
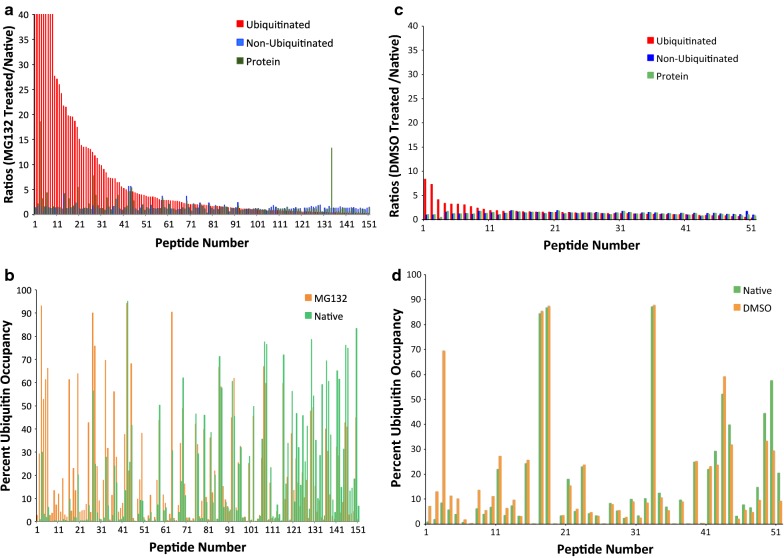
Ubiquitin occupancy, non-ubiquitin occupied and total protein ratios were generated for all partially ubiquitinated peptides detected in MG132 and DMSO control treated samples. **a** Relative ubiquitinated, non-ubiquitinated and protein ratios for all partially ubiquitinated peptides in the MG132 treated vs. native state. **b** Percent ubiquitin occupancy for partially ubiquitinated peptides in the MG132 treated and native conditions. **c** Relative ubiquitinated, non-ubiquitinated and protein ratios for all partially ubiquitinated peptides in the DMSO treated vs. native state. **d** Percent ubiquitin occupancy for partially ubiquitinated peptides in the DMSO treated and native conditions
