# Supplementary material for: Proteomic analysis of degradation ubiquitin signaling by ubiquitin occupancy changes responding to 26S proteasome inhibition
Source: Clin Proteomics. 2020 Jan 25;17:2. doi: 10.1186/s12014-020-9265-x (PMC6982382; doi:10.1186/s12014-020-9265-x)
Supplement: Supplementary file 1 — Additional file 1: Table S1. Ubiquitin occupancy of partially ubiquitinated peptides detected in the ubiquitinated and non-ubiquitin modified form in SKOV3 ovarian cancer cells after MG132 treatment. Table S2. Ubiquitinated peptides that are only detected in the modified state in ubiquitin-enriched samples following MG132 treatment of SKOV3 cells. Table S3. Ubiquitin occupancy of partially ubiquitinated peptides identified in the DMSO control treated sample. Table S4. Peptides only detected as ubiquitinated after DMSO treatment, with no corresponding non-ubiquitinated form in the global DMSO data set [file 12014_2020_9265_MOESM1_ESM.docx]

Table S1

Table S2

Table S3

Table S4
